# Understanding multifactorial drivers of child stunting reduction in *Exemplar* countries: a mixed-methods approach

**DOI:** 10.1093/ajcn/nqaa152

**Published:** 2020-07-10

**Authors:** Nadia Akseer, Tyler Vaivada, Oliver Rothschild, Kevin Ho, Zulfiqar A Bhutta

**Affiliations:** Centre for Global Child Health, Hospital for Sick Children, Toronto, Canada; Dalla Lana School of Public Health, University of Toronto, Toronto, Canada; Centre for Global Child Health, Hospital for Sick Children, Toronto, Canada; Gates Ventures, Kirkland, Washington, USA; Gates Ventures, Kirkland, Washington, USA; Centre for Global Child Health, Hospital for Sick Children, Toronto, Canada; Dalla Lana School of Public Health, University of Toronto, Toronto, Canada; Center of Excellence in Women and Child Health, the Aga Khan University, Karachi, Pakistan

**Keywords:** stunting, linear growth, children, determinants, drivers, exemplar, mixed methods, framework

## Abstract

**Background:**

Several countries have notably reduced childhood stunting relative to economic growth over the past 15–20 y. The Exemplars in Stunting Reduction project, or “*Exemplars*,” studies success factors among these countries with a lens toward replicability.

**Objectives:**

This paper details the standardized mixed-methods framework for studying determinants of childhood stunting reduction applied in *Exemplars* studies.

**Methods:**

An expert technical advisory group (TAG), criteria for identifying *Exemplar* countries, evidence-based frameworks, mixed methodologies (quantitative, qualitative, policy, literature review), effective research partnerships, case study process and timeline, and data triangulation and corroboration are presented.

**Results:**

Experts in health, nutrition, and evaluation methods were selected at the study outset to provide technical support to all phases of research (TAG). Exemplar countries were selected by the TAG, who considered quantitative data (e.g., annual rates of stunting change compared with economic growth, country population size) and qualitative insights (e.g., logistics of country work, political stability). Experienced country research partners were selected and an inception meeting with stakeholder consultations was held to launch research and garner support. Evidence-based conceptual frameworks underpinned all *Exemplars* research activities. A systematic review of published peer-reviewed and grey literature was undertaken, along with in-depth policy and program analysis of nutrition-specific and -sensitive investments. Both descriptive and advanced quantitative analysis was undertaken (e.g., equity analyses, difference-in-difference regression, Oaxaca-Blinder decomposition). Qualitative data collection using in-depth interviews and focus groups was conducted with national and community stakeholders (i.e., child care workers and mothers) to understand country experiences. The case study process was iterative, and all research outputs were triangulated to develop the stunting reduction narrative for each country. Findings were shared with country experts for weigh-in and corroboration through dissemination events.

**Conclusions:**

*Exemplars* research uses a mixed-methods framework for studying positive outliers that can be applied across diverse health and development outcomes.

## Introduction

Child growth stunting [i.e., height-for-age *z* score (HAZ) ≥2 SDs below the median] is a physical manifestation of chronic malnutrition and has been linked to higher rates of suboptimal development, morbidity, and mortality in young children, with consequences that often endure later in life (1). Given the multifactorial nature of stunting determinants, efforts to reduce the burden of stunting at the population level require interventions from both within and outside the health sector. In fact, implementation of both nutrition-sensitive and nutrition-specific policies and programs yields the greatest economic gains, with high benefit-cost ratios (2).

Globally, childhood stunting has decreased from 39.3% to 21.3% between 1990 and 2019 (3). The *Exemplars in Stunting Reduction* project, hereafter referred to as “*Exemplars*,” studied success factors among 5 countries that have achieved a rapid rate of stunting reduction relative to their economic growth. These include Peru, Kyrgyz Republic, Nepal, Senegal, and Ethiopia. Each of these countries have managed to decrease childhood stunting prevalence by almost 50% over a 15–20-y period and have shown outsized reduction as expected from economic growth alone.

Studying changes in childhood growth and stunting requires not only a multisectoral policy lens but also multifaceted methodologies. The understanding that quantitative and qualitative methodologies in isolation are inherently limited in assessing causality or studying theoretical pathways of change is the premise of mixed-methods research (4). A mixed-methods analysis approach offers a framework for combining methodologies that draws on the potential strengths of both qualitative and quantitative methods (5) and has become rapidly popular in social and behavioral science research (6). Through enabling researchers to explore diverse perspectives and relationships in a multifaceted research question (7), the mixed-methods approach aims to increase knowledge and validity in the empirical evidence to examine that question (8). Such is our motivation for adopting the mixed-methods framework when studying drivers of child stunting reduction.

Several earlier efforts have applied mixed methods when examining stunting reduction in low- and middle-income countries (LMICs) (9–16). These have yielded valuable insights into the individual, household, community, and macro drivers of change, and used a range of methods, including stakeholder interviews/group discussions, regression-based decompositions, and policy analyses (17). Our work augments existing evidence by introducing a mixed-methods framework for studying determinants of childhood stunting reduction that incorporates evidence-based frameworks, diverse and exhaustive methodologies (descriptive and causal quantitative analyses of survey datasets, administrative data and ecological variables; qualitative data collection and analyses; policy and program analyses and ranking; and systematic literature reviews), effective research partnerships, engagement with experts at all stages, and critical data triangulation and corroboration to develop a holistic narrative of change.

This methods paper introduces our standardized mixed-methods framework applied across all *Exemplars* in stunting reduction country case studies. Our framework is based on the broad *Exemplars in Global Health* approach for studies of positive outliers (**Supplementary Appendix 1**) (Carter A, Akseer N, Ho K, Rothschild O, Bose N, Binagwaho A, Hirschhorn LR, Price M, Muther K, Panjabi R et al. Methods for identifying and learning from countries that demonstrated exemplary performance in improving health outcomes and systems. Unpublished. 2020). This paper presents an overview of methods; therefore, ethical review was not required.

## Assembling a Technical Advisory Group

At the outset of the *Exemplars* project, we assembled a technical advisory group (TAG) of global experts in health, nutrition, and evaluation methods (**Supplementary Appendix 2**). Experts represented a range of sectors, including academia, donor, and nongovernmental organization (NGO) communities, and included representation from the World Bank, the Bill & Melinda Gates Foundation (BMGF) and International Food Policy Research Institute (IFPRI). The TAG was established to ensure *Exemplars* research was scientifically robust, current and relevant to diverse stakeholders, and utilizing valid cutting-edge methodologies and tools. Specifically, the TAG provided technical inputs and support through all phases of *Exemplars* research, including country selection, methodology design and implementation, and result interpretations and inferences, as well as partnered communication and dissemination activities. The TAG convened in-person or remotely multiple times throughout the year as required.

## Identifying Stunting Reduction *Exemplar* Countries

Given accelerated under-5 stunting prevalence reduction globally and in several LMICs after the year 2000, *Exemplars* largely focused on highlighting positive outliers in the 2000 to 2015 period. Our systematic country selection process aimed to identify true exemplars (i.e., those that reduced stunting prevalence beyond the projected nutritional gains associated with general economic growth) or “positive outliers.” To this end, the process of selecting case study countries involved plotting the compound annual growth rate (CAGR) in absolute stunting prevalence as a function of the CAGR in gross domestic product (GDP) per capita. We also found no difference in country ranking with sensitivity analyses using gross national income (GNI) per capita instead of GDP and average annual percentage point change (AAPC) instead of CAGR. We stratified analyses by World Bank income groups (low, lower middle, and upper middle) to compare countries within similar income bands (18). Using the plots, we identified those countries with: *1*) steep declines in stunting rates over time and/or *2*) high CAGR in stunting prevalence relative to CAGR in GDP per capita. In other words, those countries that demonstrated greater reductions in stunting prevalence while experiencing relatively smaller increases in GDP per capita were ideal candidates for case study selection.

**FIGURE 1:**
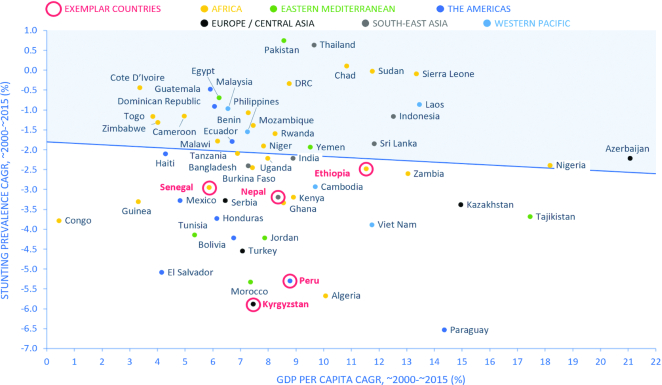
Scatterplot of the average CAGR in national-level under-5 stunting prevalence as a function of CAGR in GDP per capita for low- and middle-income countries. For the base year for each country, we used the closest year to 2000 for which a stunting estimate was available, going back no further than 1997. For the end year, we used the most recent estimate available. Matching base and end years were used for the GDP per capita estimates. CAGR refers to compound annual growth rate. CAGR, average annual rate of change; CAGR, compound annual growth rate; GDP, gross domestic product. Reproduced from Stunting, Joint Malnutrition Estimates, 2018; GDP per capita, World Bank.

**FIGURE 2:**
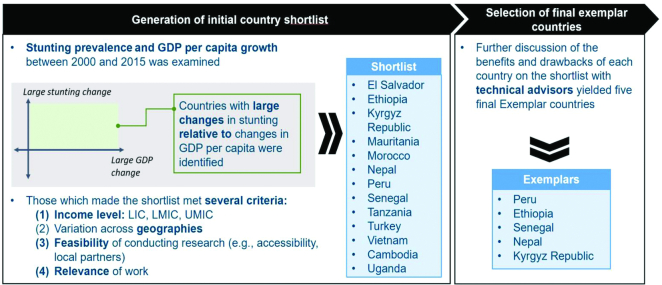
Exemplar country selection process. GDP, gross domestic product; LIC, low-income country; LMIC, low- and middle-income country; UMIC, upper middle–income country.

**Figure 1** illustrates the scatterplot for all LMICs; plots separated into income bands are shown in **Supplementary Appendix 3**. Countries ranked in the bottom left quadrant are the closest to exemplar status; a total of 13 countries were shortlisted by the TAG in this manner (Supplementary Appendix 3, **Figure 2**). The TAG also considered additional factors such as the total population of each country (minimum threshold of 5 million), variability across income bands and geographic region, physical accessibility/country security, feasibility of case study activities (e.g., qualitative data collection), and the existence of local contacts and potential partners. Using these criteria, a total of 5 countries were selected for phase 1 of the *Exemplars* project (**Figure 2**), each representing a different region of the world; i.e., Peru (Latin America), Kyrgyz Republic (Central Asia), Nepal (South Asia), Senegal (West Africa), and Ethiopia (East Africa).

## Forming Effective Research Partnerships

All case studies were led and coordinated by the Centre for Global Child Health, Hospital for Sick Children (SickKids) in Toronto, Canada. Identifying country co–principal investigators and collaborating with local research teams, however, was critical to each study. We selected academic partners with experience in diverse disciplines related to child stunting, such as public health, nutrition, maternal and child health, demography, and public finance and economics. Research partners for each *Exemplar* country are listed in **Supplementary Appendix 4**.

Our country partners were pivotal in all stages of the research; specifically, they lead the qualitative data collection and program/policy analysis, and provided support to the systematic literature review and quantitative analyses. Research partners also coordinated and facilitated an inception meeting at a central location that was attended by >20 national experts across sectors including government (health and nonhealth sector ministries), academia, UN agencies, the World Bank, the WHO, NGOs (e.g., Hellen Keller International), and frontline workers (e.g., pediatricians who work with children), among other child nutrition interest groups. These study launching events aimed to proactively engage key stakeholders in all planned aspects of the study, including but not limited to identifying datasets, locating policy and program documents, discussing methodology, identifying participants for qualitative inquiry, increasing awareness and ownership of *Exemplar* findings, and identifying strategic partnerships and opportunities for dissemination.

## Conceptual Framework

**FIGURE 3:**
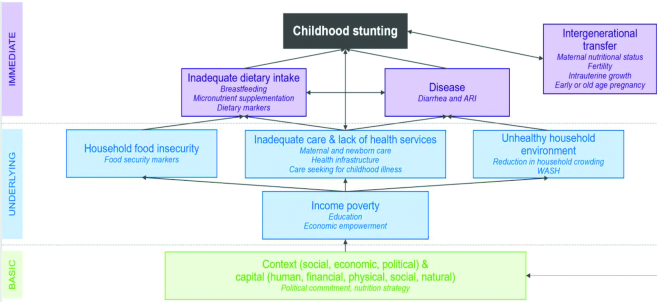
*Exemplars* conceptual framework for determinants of childhood stunting. WASH, clean water, sanitation, and hygiene,

We used the conceptual frameworks for malnutrition published by UNICEF (1990) (19) and Black et al. (2008, 2013) (1) to guide the design, analyses, and inferences across all research activities. Paper 1 of this supplement provides further detail on the selection of the frameworks and their utility in stunting research (20). The frameworks propose a causal pathway to maternal and child undernutrition operating through basic, underlying, and immediate determinants. For *Exemplars*, we adapted the framework to study determinants of childhood stunting reduction (**Figure 3**) and included linkages to nutrition-specific and sensitive interventions across sectors; see **Supplementary Appendix 5** for the example from Nepal. The adapted conceptual framework resulted from iterative discussions between the research leads and TAG, while considering project scope and data availability.

## Research Activities

Key methods used in *Exemplars* research and its purpose in our work are summarized in **Table 1**. Subsequently, we detail our methods and show illustrative country examples.

**TABLE 1:** Research methods and objective in *Exemplar's* research

Research activity	Method	Objective
Understanding the current state of evidence	Systematic literature review	Synthesize information on contextual factors, national and subnational interventions, policies, strategies, programs, and initiatives that may have contributed to reductions in child stunting in country over time Retrieve literature to inform research process from study planning and answering study objective to results interpretation
Quantitative data analysis	Geospatial analysisEquity evaluationGrowth curve analysis	Examine distribution of stunting across country and between important population subgroups to examine inequalities Assess child growth–faltering trajectories by age to understand stunting risk at birth vs. postnatally and changes over time
	Linear mixed-effect regression	Panel dataset time-series analysis using individual- and household-level and ecological data from start year to end year to understand major predictors of stunting decline
	Oaxaca–Blinder decomposition	Regression-based analysis based on individual- and household-level data to understand major predictors of stunting decline in from start year to end year
Qualitative data collection and synthesis	Focus group discussion/in-depth interviews	Understand national and community stakeholder perspectives on country's nutrition evolution (focused on progress in stunting) and the major contributing factors Access key sources of data related to financials/budget/expenditure on nutrition-specific and -sensitive initiatives
Program and policy landscape	Policy and program document review	Gain comprehensive understanding of major nutrition-specific and -sensitive policies/programs/strategies adopted and implemented at scale that may have impacted child stunting

### Understanding the current state of evidence

Each case study started with a systematic review of published and unpublished literature synthesizing information on contextual factors, national and subnational interventions, policies, strategies, programs, and initiatives that may have contributed to stunting reduction in the country. The systematic review was critical for understanding the current state of knowledge on stunting in each country, for informing study methodology and potential data sources, for identifying knowledge gaps, and for corroborating (or contrasting) our results. This step was pivotal in ensuring *Exemplars* analysis did not duplicate but rather enhanced the existing understanding of stunting in each country. We used standard systematic review methodology (see **Supplementary Appendix 6** for the Senegal example). We searched >15 online search engines (e.g., Medline, WHOLIS) and multiple grey literature sources, including government and UN websites, among others. Three broad categories of search terms were used: stunting, child, and country. We searched for literature in all languages published after 1990. Articles were title and abstract screened for relevance, and standardized inclusion and exclusion criteria were applied across countries; see **Supplementary Appendix Figure 4** for the example flow diagram from Senegal.

### Quantitative data analysis

#### Data sources

The main quantitative data sources used in *Exemplar* studies were the demographic and health surveys (DHSs) and multiple indicator cluster surveys (MICSs). These nationally representative household surveys utilize comparable, standardized methods to gather data for a wide range of indicators in the areas of population, health, and nutrition (21, 22). Our TAG concurred that the standardized methodologies in such surveys would enable objective comparisons between and within countries over time for anthropometric and determinant indicators. Original child- and household-level data were used for all analyses. To fill in data gaps, particularly for areas where there is generally a paucity of information in MICS and DHS surveys (e.g., food security and dietary intake for all under-5 children), we incorporated area-level (“ecological”) data from different data sources that aligned with the study time period. These sources differed across countries but typically included other national surveys or administrative data sources.

#### Outcomes and covariables

The main study outcomes were child HAZ and stunting calculated using WHO child growth standards (23). The hierarchical clustering of proposed determinants (24) (“covariables”) of stunting is illustrated in Figure 3, where more proximal factors function as mediators of distal factors. We organized covariables into distal, intermediate, and proximal levels to align with basic, underlying, and immediate drivers in our conceptual framework (**Table 2**). Indicators for analysis were selected from the literature and in consultation with the TAG. Variables from DHSs, MICSs, and other sources that were deemed to be of adequate quality, were valid and reliable, and were appropriate direct measures of constructs or adequate proxies were considered for the study. An expert ranking of variable “strength” based on the above criteria is shown in **Supplementary Appendix 7**. Three research experts who were well versed in biostatistics, epidemiology, and global health survey datasets (NA, KH, and ZAB) independently ranked each variable strength from 1 to 5, and an unweighted mean was calculated. Using this method, variable strength ranged from 2/5 to 5/5, with an average strength score of 3.7/5, which suggested that indicators in *Exemplars* studies were of adequate quality on average but that interpretations of analysis should be made with caution, particularly for those based on lower-ranking variables (e.g., proxies of food security, dietary intake, and low birth weight).

**TABLE 2:** Indicators available for quantitative data analysis

Level, domain, and subdomain	Indicators
Proximal	
Inadequate dietary intake domain	
Breastfeeding	Duration of breastfeeding
Micronutrient supplementation	Vitamin A supplementation, MNPs where applicable
Dietary markers	Consumption of grains, legumes, dairy, flesh foods, eggs, vitamin A–rich fruits and vegetables, other fruits and vegetables, minimum dietary diversity^1^
Disease domain	
Diarrhea and acute respiratory infection	Diarrhea and acute respiratory infection
Intermediate	
Household food insecurity domain	
Food security	Altitude, daily intake of calories, total consumable crop yield
Inadequate care and lack of health services domain	
Maternal and newborn care	SBA, ANC4+
Health infrastructure	Health workers per 10k population; health facilities per 10k population
Care seeking for childhood illness	ORT, care seeking for pneumonia
Unhealthy household environment domain	
Reduction in household crowding	Number of household members
Water, sanitation, and hygiene	Open defecation, access to piped water source, improved water source, improved sanitation
Distal	
Income poverty domain	
Education	Mother years of education, father years of education
Economic empowerment	Asset index, poverty line
Proximal	
Intergenerational transfer domain	
Fertility, early or old age pregnancy	Parity, interpregnancy interval, maternal age, adolescent birth, older mother birth
Maternal nutritional status, intrauterine growth	Maternal height, maternal BMI, anemia, low birth weight

1 These indicators were only available for 6–23 mo in DHS surveys. ANC4+, ≥4 antenatal care visits/services among pregnant women; DHS, demographic and health survey; MNP, micronutrient powders; ORT, oral rehydration therapy; SBA, skilled birth attendance.

#### Descriptive analysis

**FIGURE 4:**
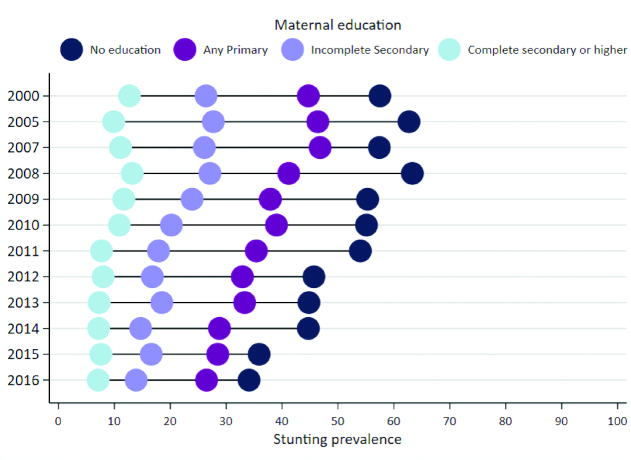
Under-5 stunting prevalence by level of maternal education in Peru, 2000–2016. Under-5, children aged <5 y. Reproduced from Huicho et al., 2020 (Peru stunting case study) (38).

Descriptive analysis methods used in *Exemplars* research are summarized in **Table 3** and detailed in **Appendix 1** (25–27, 24, 28–35). In brief, we conducted equity analyses of stunting prevalence over time by geographic region, wealth quintile, maternal education, urban/rural residence, and child gender. The slope index of inequality (SII) and concentration index (CIX) were generated to examine socioeconomic inequalities in child stunting using the entire population distribution (as opposed to only extreme wealth quintiles) (29, 30). As an example, **Figure 4** illustrates stunting prevalence by maternal education in Peru from 2000–2016; inferences from such analyses included *1*) analyzing the gap (e.g., increase or decrease) between extreme groups over time; *2*) stunting prevalence for the least educated, most educated, or middle groups; and *3*) changes in stunting prevalence for any group over time. We analyzed both relative and absolute changes in stunting prevalence for each geographic region by calculating the compound annual growth rate and AAPC, respectively. Both metrics provide value and selection of one compared with the other should depend on research interest. In Peru, for instance, departments (i.e., provinces) that had the highest reduction in stunting prevalence according to CAGR were slightly different than those identified with AAPC; our selection of “top performing” regions was based on a composite of those that ranked high in absolute and relative change.

**TABLE 3:** Descriptive analysis methods used in *Exemplar's* research

Purpose and methods	Summary
Equity analysis^1^	
Disaggregation by subnational geography, wealth quintile, maternal education, urban vs rural residence, and child gender	Estimates of stunting prevalence for key subpopulation
SII and CIX	Measure absolute and relative wealth inequalities, respectively
CIX	Estimated from logistic regression models of the cumulative distribution of the asset index, plotted against stunting prevalence
5 × 5–km geospatial estimates	Modeled estimates that use location data to estimate the subnational distribution of stunting prevalence at 5 × 5–km granularity
Rates of reduction^2^	
CAGR	Assessed relative change (decline) in stunting prevalence over time for each geographic region
AAPC	Estimated through ordinary least square regression models; stunting prevalence regressed on survey year
Population shifts in growth faltering	
Victora curves	Smoothed local polynomial regressions to depict HAZ vs. child age (in months) predictions with 95% CIs, estimated by surveys (e.g., DHS or MICS)
HAZ kernel density plots	Depict the distribution of child HAZ scores and enable assessment of skewness and kurtosis; stratified by child age groups: <6, 6–23, or ≥24 mo

1 Analysis accounts for survey design and weighting. AAPC, average annual percentage point change; CAGR, compound annual growth rate; CIX, concentration index; DHS, demographic and health surveys; HAZ, height-for-age *z*-score; MICS multiple indicator cluster surveys; SII, slope index of inequality.

^2^Analyses conducted to show changes over survey round.

We generated child HAZ kernel density plots for all under-5 children and stratified for children aged <6, 6–23, and >24 mo. Such analyses enabled inferences regarding *1*) whole-population shifts in HAZ plot; *2*) changes in mean HAZ across surveys, to examine whether reductions in growth faltering were occurring for the entire spectrum of children in that age group; and *3*) changes in skewness and kurtosis of the curve, to explore inequalities in the growth-faltering spectrum. See example illustrations for Ethiopia in **Supplementary Appendix 8**.

**FIGURE 5:**
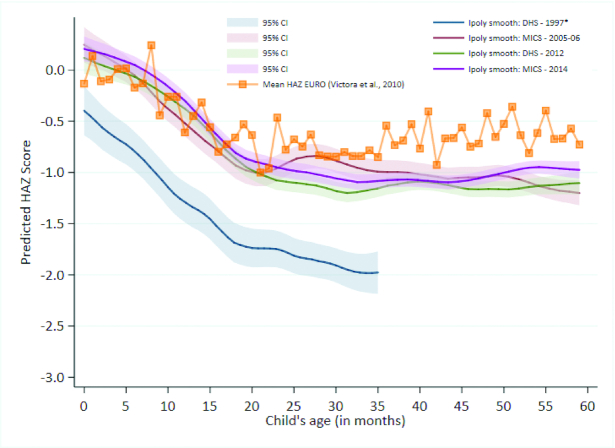
Victora curves for under-5 children in the Kyrgyz Republic, 1997–2014. *Survey only sampled children under-3; under-5 stunting prevalence was extrapolated using: percentage under-5 stunted = −0.0114274 + (1.104429 * percentage under-3 stunted) (41). WHO Regional Office for Europe (EURO) mean. Under-5, children aged <5 y. Reproduced from Wigle et al., 2020 (Kyrgyz Republic stunting case study) (42).

Child growth curves, or “Victora curves,” show predicted child HAZ that has been estimated from smoothed local polynomial regressions plotted against child age (36, 37). See **Figure 5** for the example from the Kyrgyz Republic. These curves allow for the examination of the growth-faltering process from birth to 5 y of age. Two crucial features of child undernutrition are revealed in Victora curves, *1*) the curve's intercept, which reveals the intergenerational susceptibility of child undernutrition or the extent to which a mother's nutrition leads to small babies at birth; and *2*) the postnatal growth-faltering process, which is typically steepest between 6 and 24 mo, after which it tapers off. Figure 5 suggests that among Kyrgyz children, stunting decline from 1997 to 2014 may be attributable to improved maternal care and nutrition resulting in reduced intrauterine growth restriction and greater child HAZ at birth, improvements in breastfeeding practices for children <6 mo old (i.e., flatter slope over time), and some benefits in disease prevention and dietary intake of children 6–23 mo old. To quantify inflection points and growth rates (i.e., slopes), we also fitted piecewise linear splines; results are shown in papers for individual countries in this supplement.

#### Multivariable analysis

Each *Exemplars* study undertook 2 sets of complementary multivariable analyses that aimed to answer the same research question (i.e., what are the main predictors of change in child linear growth in a country during critical time periods?). The linear regression based on panel datasets uses a difference-in-difference analysis framework in which interactions of time with covariables are used to assess factors impacting HAZ decline (27). This process allows for the analysis of multiple years of survey data and adjusts for baseline levels of covariables and varying hypothesized growth trajectories through the interaction terms. We also used Oaxaca–Blinder decomposition methods (16, 39) to assess determinants of nutritional change over time in each country. These methods based on individual-level data have high statistical power and have been widely used to assess nutrition determinants in LMIC settings (14–16, 40). The Oaxaca–Blinder decomposition is based on the same set of individual- and household-level data (with ecological variables). However, by design, the decomposition only uses 2 survey time points in a given analysis and thus “ignores” in-between survey rounds and any intermittent fluctuations in the predictors. As has been suggested in previous decomposition analyses, we operationalize child HAZ as the linear growth outcome due to its greater statistical efficiency relative to the dichotomous child-stunting variable. Each of the 2 multivariable regression–based analysis methods has its own strengths and limitations. However, as sensitivity analyses, study inferences should be anchored in both, and congruent findings between the methods strengthen the key messages. See **Appendix 2** for detail on how these methods were implemented in *Exemplars* studies.

**FIGURE 6:**
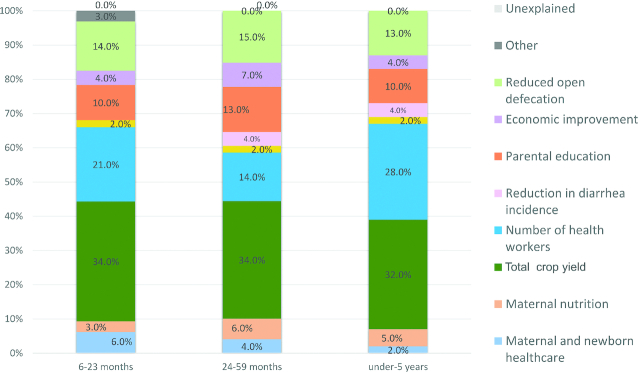
Oaxaca–Blinder decomposition of determinants of mean child HAZ change in Ethiopia by age group, 2000–2016. Parental education breakdown: children 6–23 mo: maternal 3.3%, paternal 3.8%; children 24–59 mo: maternal 7.5%, paternal 0%; children under-5: maternal 4.6%, paternal 3.6%. For the 6–23–mo age category delivery in medical facility is included in maternal and newborn healthcare (6.7%). Other categories include child age, gender, and region for all age groups in addition to maternal age (0.5%) for the 6–23–mo old age group. Reproduced from Tasic et al, 2020 (Ethiopia stunting case study) (45).

To illustrate, **Supplementary Appendix 9** shows the difference-in-difference model results for maternal education predicting child HAZ change in Senegal from 1992 to 2017, after adjusting for confounders. Here, the statistically significant interaction term between the survey year and maternal education was visualized using margins plots. Evidently, the mean HAZ trajectory differs for mothers with no education, 1–5 y of education, and >5 y of education, which suggests that maternal education was a statistical driver of change in HAZ from 1992 to 2017. **Figure 6** shows HAZ decomposition results for Ethiopia by age group. These results reveal that major drivers of HAZ improvement across age groups were largely the same; i.e., increases in total consumable crop yield (between 32% and 34%), greater number of health workers (14–28%), reduction in open defecation (13–15%), and improvements in parental education (10–13%).

#### Qualitative data collection and synthesis

The qualitative component of *Exemplars* studies aimed to increase understanding of the determinants of stunting reduction through exploring the perspectives of key national stakeholders in the development and implementation of relevant policies and programs and the experiences of child care workers and mothers at the community level. The specific objectives for each country were the following: *1*) to identify the nutrition-specific and -sensitive key events (policies, strategies, laws, legislation, and programs) that may have contributed to a reduction in child stunting; *2*) to understand the main success factors and challenges of relevant nutrition-specific and -sensitive facilitators of key events (policies, strategies, laws, legislation, and programs); *3*) to identify important contextual factors that have functioned as enablers or drivers of national-level stunting change; and *4*) to document community-level perspectives and experiences on the stunting transition by consulting childcare workers and mothers of young children.

We undertook 3 independent research activities to inform study objectives. At the first stage, national stakeholders (e.g., government policymakers) were interviewed to provide insight and expertise on objectives 1, 2, and 3. This top-down approach aimed to solicit macro-level perspectives and experiences in health and nutrition in each country. To understand how individuals in the community received and implemented major nutrition-specific and -sensitive policy and program events, as well as their experiences in the nutritional transition as a whole, we consulted childcare workers in the community (e.g., at schools, health facilities, etc.) and the mothers of young children. These latter 2 research activities largely informed objectives 3 and 4 and also shed light on objective 2.

Primary qualitative data collection methods involved conducting in-depth interviews (IDI) with key informants at national and community levels (10–20 participants), and focus group discussions (FGD) with mothers in communities (2–12 focus groups with 10–15 participants each). Our conceptual framework informed design of the IDI and FGD guides, as well as analysis and interpretation of the qualitative data. Our qualitative data collection tools were also informed by existing literature and nutrition questionnaires; for example, the IFPRI's nutrition-focused qualitative data collection toolkit was consulted and relevant tools were adapted to our research objectives as appropriate (43). Our approach for consulting mothers was based on previously published designs and focused on identifying key nutrition-related individual, household, and environmental factors that changed over time by interviewing older mothers (i.e., mothers with under-5 children 15–20 y ago) and younger mothers (i.e., mothers with current under-5 children) (44). Data collection tools used in the Ethiopia case study are included as **Supplementary Appendix 10**. Data were analyzed using thematic analysis aligning with the basic, underlying, and immediate causes of child undernutrition. Methods detailed for the *Exemplars* studies are provided in the respective papers in this supplement.

#### Policy and program landscape

**FIGURE 7:**
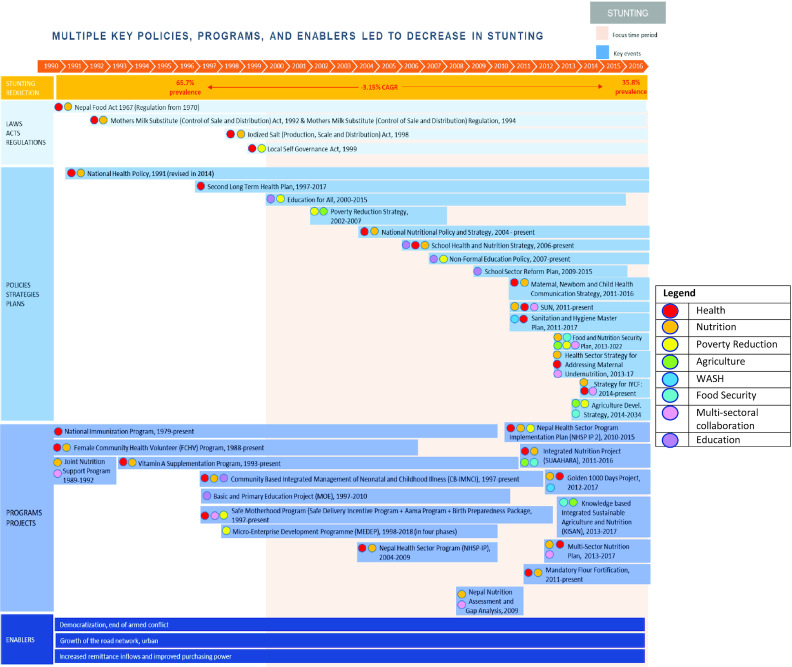
Overview of laws, policies, programs, and enablers of stunting reduction in Nepal from 1990–2016. Entries that are associated with one another, or laws/policies that resulted in a program are indicated in the same shade of blue.

We assembled a timeline of key nutrition-specific and -sensitive laws, acts, and regulations; policies, strategies, and plans; and programs in each country through an iterative approach drawing on several of the above methods. Starting with a desk review of literature identified through our systematic approach, a suggested timeline was proposed by SickKids researchers and the country research team. This timeline was shared with national expert stakeholders to obtain their corroboration and insight on any missing initiatives. After reviewing additional literature and specific policy and program documents as suggested by experts, a second iteration of the timeline was proposed. This process ensued until consensus was reached between all research partners and experts. For each initiative, we thoroughly examined published and unpublished documents to track down the information displayed in **Table 4**. Collectively, all information was subsequently used to rank items by level of importance (i.e., very important, important, likely important, not important, and promising recent initiative) to stunting reduction in each country. The timeline was generally 1990–2018, but also spanned earlier decades in countries where these time periods were deemed relevant to stunting decline. We also attempted to track financial data linked to the timeline with the aim of tagging dollar amounts for financial allocations and actual disbursements and budgets and expenditures of the various programs, policies, interventions, and other initiatives. The scan for financial commitments and spending spanned many sectors, including government, development partners, NGOs, and others as applicable. **Figure 7** shows the final timeline for the Nepal case study and highlights the contribution of efforts from health, nutrition, poverty reduction, agriculture, safe drinking water, sanitation, hygiene, food security, education, and broad multisectoral action. The final policy and program timelines for *Exemplars* studies are provided in the respective papers in this supplement.

**TABLE 4:** Components of each policy or programmatic initiative

Description An overview of the major objectives of the program/policy Area of the country where the program/policy was delivered Population reached (number of people reached, setting) Details of scale-up Delivery platform: the channel by which a intervention reaches the population in need. Fortification-based platforms Financial incentive-based platforms Community-based platforms School-based platforms Technology-based platforms Key stakeholders Initiation process Key components Monitoring and evaluation of implementation Funding Success factors/barriers

## Evidence Triangulation

A final and critical step of *Exemplars* studies was to iteratively use data from each research activity to derive and contextualize key results. Evidence-based inferences supported by our analyses were first proposed by SickKids research leads, and then subsequent iterations were added on with country partners and subject matter experts and contrasted with the literature to develop the final narrative. This thorough process of weighing the existing literature with empirical data and analysis and expert insight ensured that inferences on causality and determined pathways of change were plausible and rooted in rigorous analysis.

## Case Study Process and Iteration

Though the 4 research activities used in *Exemplar* studies have been presented sequentially in this paper, it should be noted that, in practice, activities occurred in tandem. This parallel approach was effective in honing our methodology as we used emergent results from one research activity to inform processes and inferences of others. Similarly, learnings from earlier case studies performed in earlier countries were used to streamline methods for subsequent studies. In general, case studies ensued as follows: *1*) preparation of preliminary literature review, policy and program snapshot, and descriptive quantitative analysis; *2*) attendance at a study inception meeting in each country to present the study background and approach and consult key stakeholders; *3*) developing and implementing methods for systematic review and qualitative, quantitative, and policy and program review work; *4*) completion of research and development of final study results; and *5*) sharing of findings and country expert consultation to iterate on inferences. Each *Exemplar* research study took about 1 to 1.5 y to complete, and all 5 phase 1 studies were completed between 2017 and 2019.

## Strengths and Limitations

**FIGURE 8:**
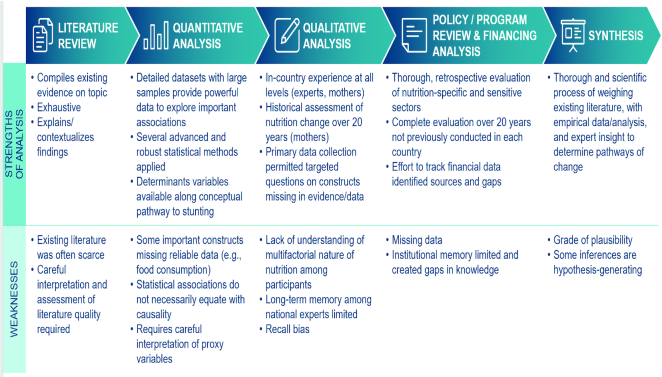
Strengths and limitations of *Exemplar's* research.

The *Exemplars* approach exercised a range of activities, including a systematic literature review, qualitative inquiry with diverse national- and community-level stakeholders, robust quantitative analyses, and policy and program mapping and an exploration of financial allocations, to paint a holistic picture of stunting decline in each country. Though mixed-methods research for studying determinants and drivers of nutritional improvements is not new, our approach improves upon the existing literature in several ways (**Figure 8**). First, the standardized lens applied across countries not only enables rigorous inferences at the country level but also permits objective comparisons across countries. Second, the range of determinants previously published was expanded by adding individual, household, and ecological variables in line with an evidence-based conceptual framework, thereby providing further granularity to our inferences. Third, the DHS and MICS surveys conducted at different time points were harmonized to permit pseudo longitudinal analyses. This allowed us to explore different time periods throughout 1990–2018, such as those spanning several peaceful democratic transitions, political commitment toward achieving the MDGs, and substantial prioritization of cross- and multisectoral nutrition-focused efforts. Fourth, the hierarchical modeling approaches undertaken in all quantitative analyses enabled more appropriate modeling of pathways (adjusting for confounders and examining mediators) between potential stunting determinants. Fifth, the qualitative component aimed to captured diverse, multilevel perspectives, particularly with the addition of focus group discussions with mothers, to highlight socioeconomic, behavior, health, and nutrition changes at the household level. Finally, this was to our knowledge the first effort that included the conduction of systematic literature reviews to compile all nutrition-relevant published and unpublished literature and an accompanying in-depth policy and program analysis for *Exemplar* countries.

Several study limitations should also be noted (Figure 8). Data pertaining to direct measures of food insecurity and dietary intake of all under-5 children (e.g., exclusive breastfeeding, appropriate introduction of complementary feeding, actual food consumption frequency and diversity) were generally lacking. As a result, we relied on proxies and available survey indicators (e.g., 24-hour recall)—though these imprecise indicators are not without their shortcomings. Information on migration patterns within countries was also limited. The DHS lacked data to measure intrauterine growth, such as low birth weight (for which data were collected only in select surveys and are poorly reported), small for gestational age babies, preterm birth, etc. Maternal height and BMI data were not available for all of our surveys of interest, and thus we could not quantitatively examine the association between these indicators and child HAZ gains for all countries. The quality of anthropometry data was of particular concern in some *Exemplar* countries, particularly Senegal, where ≥1 DHS survey (2010–2011) had child anthropometry data with >20% flagged/implausible values. Paper 3 of this supplement sheds additional light on anthropometry quality and metrics for its evaluation.

There was generally a limited scope of ecological variables available for our assessment, and several important domains were not quantifiable even through area-level predictors, like food security, agriculture production, etc.

We were not able to quantitatively examine several drivers due to the challenges posed in measuring such phenomena (e.g., political will, political instability, changes in governance structures, and conflict) and limitations of data availability for several programs (e.g., vitamin A supplementation, community health workers/volunteers, essential package of health services). Our mapping of financial data pertaining to nutrition-specific and -sensitive initiatives may be incomplete, considering that we reported only data we could obtain from stakeholders and the published literature. Significant limitations in national health expenditure data and the financial allocations for specific programs highlight a gap in our understanding of the investment, implementation, and scale of these efforts.

We were also not able to conduct age-stratified analyses for all countries, particularly for children <6 mo of age, due to limited statistical power and/or meaningful HAZ change over time in these populations. Thus, we could have missed important age-specific HAZ determinants (e.g., breastfeeding and dietary intake from food-frequency questions). Another limitation pertains to confounding. Although we adjusted for confounding variables in the quantitative analyses, some residual confounding may remain from unmeasured confounders or poorly estimated variables. Interactions between different factors within and between the different hierarchical levels may also affect the results, although due to measurement challenges they were not assessed. Given the reliance on cross-sectional survey datasets for quantitative analyses, causal inference was challenging and interpretations had to be made with caution.

Participant recall, high staff turnover, and poor institutional memory were also limitations of the qualitative inquiry.

## Conclusion

This paper presented the *Exemplars* mixed-methods approach for studying determinants of childhood stunting decline over a 15–20-y period in countries that have made significant progress. Despite limitations in data, the approach brings several strengths for building a comprehensive and rigorous narrative of change. In scaling up efforts to reduce morbidity and premature mortality among mothers and children globally, we hope such mixed-methods research approaches can become commonplace in studying health and development outcomes.

## Supplementary Material

nqaa152_Supplemental_File

## Notes

Funded by a grant to the Centre for Global Child Health from Gates Ventures. The funder had no role in the design, implementation, analysis, or interpretation of the data.

Data described in the manuscript, code book, and analytic code will be made available upon request pending application and approval.

**Disclosure Statement: Published in a supplement to the *American Journal of Clinical Nutrition*. Publication costs for this supplement were defrayed in part by the payment of page charges by Gates Ventures. The opinions expressed in this publication are those of the authors and are not attributable to the sponsors or the publisher, Editor, or Editorial Board of the *American Journal of Clinical Nutrition*.

The Guest Editor for this supplement was Mark Manary. Supplement Coordinator disclosure: no conflicts to disclose. Supplement Coordinator for the supplement publication was Nadia Akseer, Gates Ventures/Hospital for Sick Children, Toronto, Canada. Supplement Coordinator disclosure: no conflicts to disclose. The Stunting Exemplars research Principal Investigator was Zulfiqar A Bhutta, Hospital for Sick Children, Toronto, Canada. Principal Investigator disclosure: no conflicts to disclose.

Supplementary Methods and Appendixes 1–10 are available from the “Supplementary data” link in the online posting of the article and from the same link in the online table of contents at https://academic.oup.com/ajcn/.

Abbreviations used: AAPC, average annual percentage point change; BMGF, Bill and Melinda Gates Foundation; CAGR, compound annual growth rate; CIX, concentration index; DHS, demographic and health survey; FGD, focused group discussions; GDP, gross domestic product; GNI, gross national income; HAZ, height-for-age *z*-score; IDI, in-depth interviews; IFPRI, International Food Policy Research Institute; LMIC, low- and middle-income country; MDG, millennium development goals; MICS multiple indicator cluster survey; NGO, nongovernmental organization; SII, slope index of inequality; TAG, technical advisory group; aged <5 y, under-5, aged <5 y.
