# Supplementary material for: Understanding multifactorial drivers of child stunting reduction in Exemplar countries: a mixed-methods approach
Source: Am J Clin Nutr. 2020 Jul 10;112(Suppl 2):792S–805S. doi: 10.1093/ajcn/nqaa152 (PMC7487431; doi:10.1093/ajcn/nqaa152)
Supplement: nqaa152_Supplemental_File [file nqaa152_supplemental_file.docx]

**Title**: Understanding multifactorial drivers of child stunting reduction in Exemplar countries: a mixed-methods approach

**Author Names**: Dr. Nadia Akseer, Tyler Vaivada, Dr. Oliver Rothschild, Kevin Ho, Dr. Zulfiqar A. Bhutta

**Supplementary Appendices**

[Supplementary Appendix 1: Background 3](#_Toc39848293)

[**Supplementary Appendix Figure 1:** Exemplars in Global Health research approach 3](#_Toc39848294)

[Supplementary Appendix 2: Technical Advisory Group 3](#_Toc39848295)

[**Supplementary Appendix Table 1:** Stunting Reduction Exemplars Technical Advisory Group 3](#_Toc39848296)

[Supplementary Appendix 3: Exemplar Country Selection 4](#_Toc39848297)

[**Supplementary Appendix Figure 2A:** Scatterplot of the average AARC in national-level under-5 stunting prevalence as a function of AARC in GDP per capita for **low-income countries**. 4](#_Toc39848298)

[**Supplementary Appendix Figure 2B:** Scatterplot of the average AARC in national-level under-5 stunting prevalence as a function of AARC in GDP per capita for **lower middle-income countries.** 4](#_Toc39848299)

[**Supplementary Appendix Figure 2C:** Scatterplot of the average AARC in national-level under-5 stunting prevalence as a function of AARC in GDP per capita for **upper middle-income countries.** 5](#_Toc39848300)

[**Supplementary Appendix Table 2:** Final shortlist of top ten countries considered for case study selection, stratified by region and income level 5](#_Toc39848301)

[Supplementary Appendix 4: Exemplar Country Partners 6](#_Toc39848302)

[**Supplementary Appendix Table 3:** Research Partners by Exemplar Country 6](#_Toc39848303)

[Supplementary Appendix 5: Example Conceptual Framework 7](#_Toc39848304)

[**Supplementary Appendix Figure 3:** Adapted conceptual framework for analyzing determinants of child undernutrition in Exemplar studies (Nepal example) 7](#_Toc39848305)

[Supplementary Appendix 6: Systematic Review Methodology (Senegal Example) 8](#_Toc39848306)

[**Supplementary Appendix Figure 4:** Literature review flow diagram 9](#_Toc39848307)

[Supplementary Appendix 7: Strength of Proxy Variables 10](#_Toc39848308)

[**Supplementary Appendix Table 4:** Expert ranking of strength of proxy variables 10](#_Toc39848309)

[Supplementary Appendix 8: HAZ Kernel Density Analysis (Ethiopia Example) 11](#_Toc39848310)

[**Supplementary Appendix Figure 4:** Kernel density plot for HAZ distribution in children <5 years in Ethiopia, DHS 2000, 2005 2011, 2016 11](#_Toc39848311)

[Supplementary Appendix 9: Difference-in-difference analysis margins plot (Senegal Example) 12](#_Toc39848312)

[**Supplementary Appendix Figure 5:** Multivariable difference-in-difference results of maternal education predicting child HAZ in Senegal, 1992-2017 12](#_Toc39848313)

[Supplementary Appendix 10: Qualitative Data Collection Tools (Ethiopia Example) 13](#_Toc39848314)

# Supplementary Appendix 1: Background

## **Supplementary Appendix Figure 1:** Exemplars in Global Health research approach


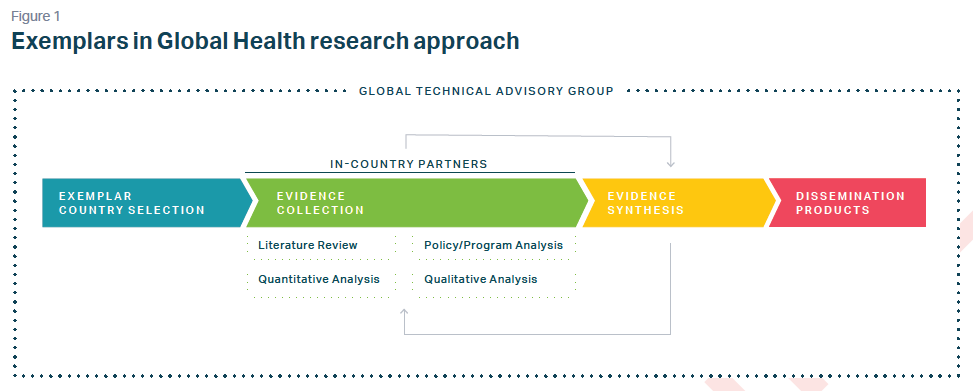


Source: Carter et al 2019, unpublished

# Supplementary Appendix 2: Technical Advisory Group

## **Supplementary Appendix Table 1:** Stunting Reduction Exemplars Technical Advisory Group

| **Advisor** | **Institution** | **Sector** |
| --- | --- | --- |
| Dr. Zulfiqar A Bhutta | Hospital for Sick Children, Toronto, Canada  Aga Khan University, Karachi, Pakistan | Academia |
| Dr. Robert E Black | Johns Hopkins University, Baltimore, MD, USA | Academia |
| Dr. Joanne Katz | Johns Hopkins University, Baltimore, MD, USA | Academia |
| Dr. Sue Horton | University of Waterloo, Waterloo, Canada | Academia |
| Dr. Cesar Victora | Federal University of Pelotas, Pelotas, Brazil | Academia |
| Shawn Baker | Former Bill & Melinda Gates Foundation | Donor |
| Dr. Ellen Piwoz | Bill & Melinda Gates Foundation | Donor |
| Dr. Meera Shekar | The World Bank, Washington, DC, USA | Donor |
| Dr. Purnima Menon | International Food Policy Research Institute, Washington, DC, USA | NGO |

# Supplementary Appendix 3: Exemplar Country Selection

## **Supplementary Appendix Figure 2A:** Scatterplot of the average AARC in national-level under-5 stunting prevalence as a function of AARC in GDP per capita for **low-income countries**.

## **Supplementary Appendix Figure 2B:** Scatterplot of the average AARC in national-level under-5 stunting prevalence as a function of AARC in GDP per capita for **lower middle-income countries.**


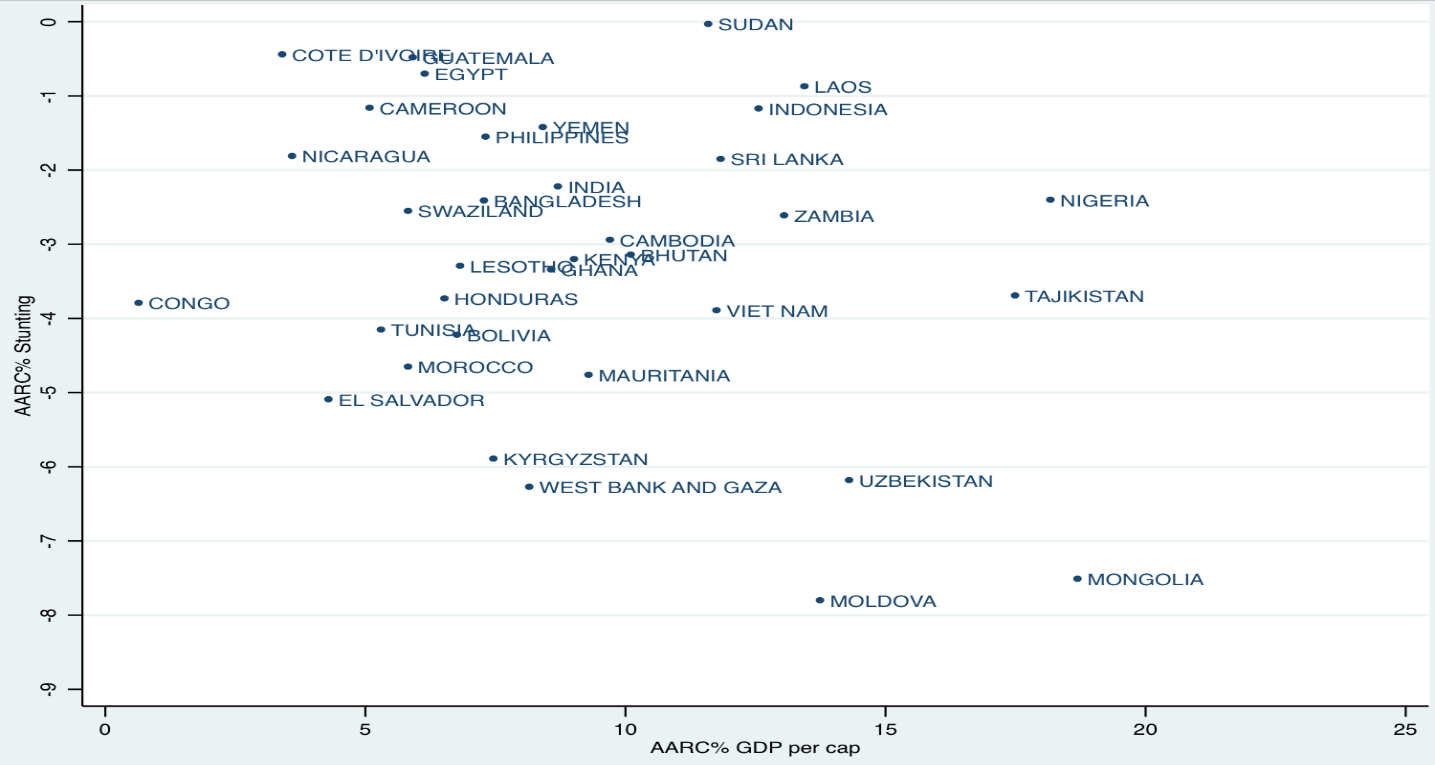


## **Supplementary Appendix Figure 2C:** Scatterplot of the average AARC in national-level under-5 stunting prevalence as a function of AARC in GDP per capita for **upper middle-income countries.**

## **Supplementary Appendix Table 2:** Final shortlist of top ten countries considered for case study selection, stratified by region and income level

|  | **Latin America** | **North Africa** | **Sub-Saharan Africa** | **South & Central Asia** | **Southeast Asia** |
| --- | --- | --- | --- | --- | --- |
| **Low income** |  |  | • Senegal  • Tanzania  • Ethiopia  • Uganda** | • Nepal |  |
| **Lower middle income** | • El Salvador | • Morocco  • Mauritania |  | • Kyrgyzstan | • Vietnam*  • Cambodia** |
| **Upper middle income** | • Peru |  |  | • Turkey |  |
| * Vietnam was selected as a potential exemplar to represent Southeast Asia, but did not appear in the original shortlist of ten countries.  **Cambodia and Uganda were added based on further technical expert deliberations | | | | | |

# Supplementary Appendix 4: Exemplar Country Partners

## **Supplementary Appendix Table 3:** Research Partners by Exemplar Country

| **Country** | **In-country lead** | **Affiliation** |
| --- | --- | --- |
| Peru | Dr. Luis Huicho | Centro de Investigación para el Desarrollo Integral y Sostenible; Centro de Investigación en Salud Materna e Infantil; School of Medicine, Universidad Peruana Cayetano Heredia, Lima, Peru |
| Kyrgyz Republic | Dr. Roman Mogilevskii | Institute of Public Policy and Administration, University of Central Asia, Bishkek, Kyrgyz Republic |
| Nepal | Dr. Mahesh Maskey | Nepal Public Health Foundation, Kathmandu, Nepal |
| Senegal | Dr. Mohamadou Sall | l'Institut des Populations de la Démographie et Santé de la Reproduction (IPDSR), Université Cheikh Anta Diop, Dakar, Senegal |
| Ethiopia | Dr. Seifu Hagos | Department of Nutrition and Dietetics, School of Public Health, Addis Ababa University, Addis Ababa, Ethiopia |

# Supplementary Appendix 5: Example Conceptual Framework

## **Supplementary Appendix Figure 3:** Adapted conceptual framework for analyzing determinants of child undernutrition in Exemplar studies (Nepal example)


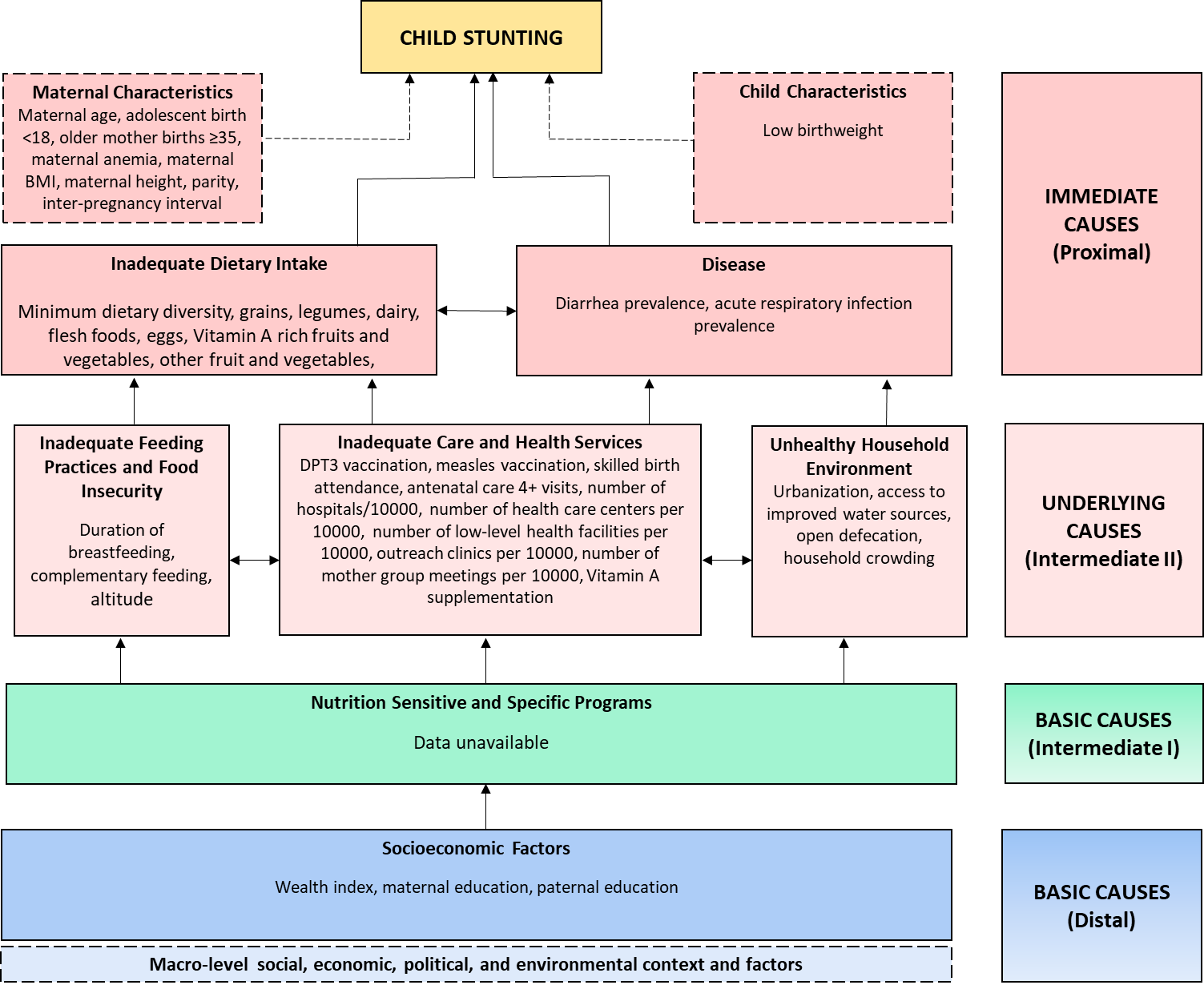


Note: framework reflects only indicators that were measurable and available for quantitative analysis.

*Altitude, birthweight and vitamin A not available in 2001 survey

Variables that were available for analysis are categorized for hierarchical analysis as follows:

- **Distal level (basic causes):** Socioeconomic factors
- **Intermediate level (underlying causes)**: Unhealthy household environment, inadequate care and health services, and inadequate feeding practices and food insecurity
- **Proximal level (immediate causes):** Disease and inadequate dietary intake. The proximal level also includes child and maternal characteristics, which are especially important to track factors that directly impact the child’s nutritional status. For example, intergenerational transmission of malnutrition and epigenetic effects, which could be measured by proxies such as low birthweight, maternal height and body mass index (BMI) amongst others.

# Supplementary Appendix 6: Systematic Review Methodology (Senegal Example)

A systematic search of published peer-reviewed and grey literature related in Senegal was undertaken in order to synthesize information on contextual factors, national and subnational interventions, policies, strategies, programs, and initiatives that may have theoretically contributed to reductions in child stunting in Senegal over time. Three broad categories of search terms were used: stunting, child, and Senegal. Keywords representing these terms were combined with Boolean operators, adapted with appropriate syntax, and executed in multiple databases. An example of a search syntax is provided below:

1. *Stunting*: "stunting" or "linear growth" or "linear growth stunting" or "HAZ" or "height" or "height-for-age" or "LAZ" or "length" or "length-for-age" or "undernutrition" or "malnutrition" or "nutr*"
2. *Child*: “child*” or “infan*”
3. “Senegal*"
4. 1 AND 2 AND 3

The search for indexed literature was conducted in 15 online databases: MEDLINE, Embase, AMED, CAB Abstracts, CINAHL, Cochrane CENTRAL, Campbell Collaboration, EPPI Centre Trials Register (TRoPHI), 3ie, JOLIS, African Journals Online, WHOLIS, LILACS, Scopus, and Web of Science. Additional searches for grey literature were conducted using Google, a hand search of reference lists of relevant reviews, and direct searching organizational websites, including: National, regional and headquarter websites for UNICEF, WHO,  UNDP, WFP, FAO, World Bank Group Open Knowledge Repository, Nutritional International, Global Alliance for Improved Nutrition, International Food Policy Research Institute, Government of Senegal including the Ministry of Health and Social Action, and Ministry of Agriculture and Rural Development.

The exported set of records were de-duplicated and screened for relevance. Records were included if they met all of the following inclusion criteria:

1. included an under-5 population in Senegal;
2. published between 1990-2017;
3. examined one or more of the determinants of chronic undernutrition (e.g. determinants, risk factors, policies, programs, interventions, or initiatives); and
4. examined effects on child growth or a reduction in stunting

Initial database searches returned 2,718 records, which was reduced to 1,728 after de-deduplication. Applying the screening criteria to titles and abstracts left 81 records, which was then reduced to 44 upon full-text review. All included studies were categorized for the purposes of thematic exploration and mapping. Three broad categories of studies were identified: i) quantitative analysis of child health or nutrition outcomes; ii) qualitative studies with quantitative analysis; and iii) grey literature reports from non-governmental and multilateral organizations. See Figure 6 for a flow diagram outlining the components and progression of the review. Relevant literature was iteratively synthesized and summarized to inform our research questions and to contrast our findings with existing evidence.

## **Supplementary Appendix Figure 4:** Literature review flow diagram


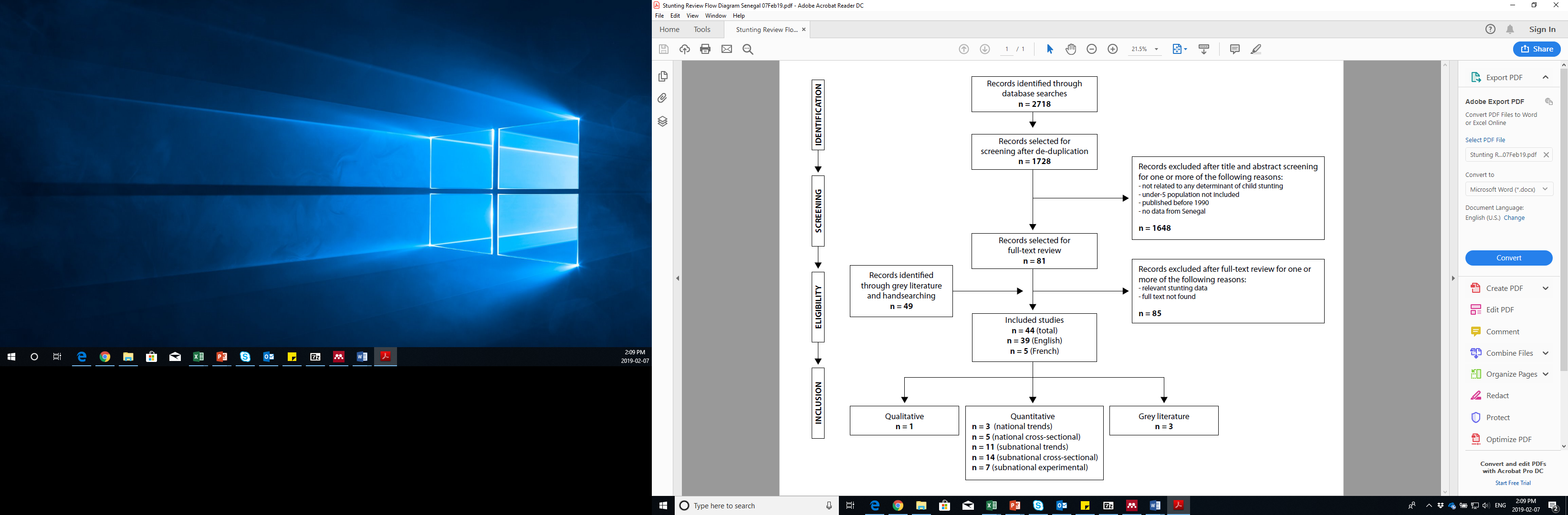


Targeted additional searches were completed for key topics to supplement and expand on important factors in the Senegal stunting narrative; a total of 217 additional documents spanning grey literature and published peer-reviewed reports were collated and summarized.

# Supplementary Appendix 7: Strength of Proxy Variables

## **Supplementary Appendix Table 4:** Expert ranking of strength of proxy variables

|  | **Driver of stunting** | **Ideal data to analyze** | **data used (individual, household, or ecological)** | **Strength of proxy (/5)** |
| --- | --- | --- | --- | --- |
| Basic | Economic/household wealth | Household expenditure | Asset index (household), poverty line (ecological) | 4 |
|  | Maternal empowerment | Validated empowerment index, decision-making, purchasing power, financial autonomy, social autonomy | Mother years of education (individual), parity (individual), interpregnancy interval (individual), age at marriage (individual) | 3 |
| Underlying | Reduction in household crowding | N/A | Number of household members (individual) | 5 |
|  | WASH | Water quality, sanitation (N/A) | Open defecation (individual), improved water source (household), piped water (household), improved sanitation (household) | 4 |
|  | Health infrastructure | N/A | Doctors per 10K (ecological), nurses and midwives per 10K (ecological), community health workers per 10K (ecological), health facilities per 10K (ecological) | 5 |
|  | MNCH | Effective coverage of interventions (e.g., quality) | SBA (individual), ANC4+ (individual) | 3.5 |
|  | Micronutrient supplementation / fortification | Coverage and compliance data on micronutrient supplementation programs | Vitamin A coverage (individual), MNP program exposure as applicable | 2.5 |
|  | Care seeking for childhood illness | Good quality HMIS or DHIS | ORT (individual), care seeking for pneumonia (individual) | 4 |
|  | Food security | Household-level food consumption | Altitude (household), daily intake of calories (ecological), consumable crop yield (ecological) | 2.5 |
| Immediate | Disease (diarrhea and ARI) | Disease-specific information (referrals, hospitalizations) from DHIS | Diarrhea in last 2 weeks (individual), ARI infection in last 2 weeks (individual) | 4 |
|  | Maternal nutritional status | Iron-deficiency anemia, micronutrient status, weight gain in pregnancy | Height (individual), BMI (individual), Anemia (individual) | 4 |
|  | Breastfeeding | Individual-level duration of breastfeeding; exclusive breastfeeding | Duration of breastfeeding | 4 |
|  | Dietary intake | Child dietary consumption by food group | Consumption of grains, legumes, dairy, flesh foods, eggs, Vitamin A rich fruits and vegetables, other fruits and vegetables, minimum dietary diversity; for children 6-23 months, based on food recall surveys | 2.5 |
|  | Fertility | N/A | Parity (individual), Inter-pregnancy interval (individual) | 5 |
|  | Intrauterine growth | Small-for-gestational age, pre-term births, weight gain in pregnancy | Low birthweight (individual) | 2 |

# Supplementary Appendix 8: HAZ Kernel Density Analysis (Ethiopia Example)

## **Supplementary Appendix Figure 4:** Kernel density plot for HAZ distribution in children <5 years in Ethiopia, DHS 2000, 2005 2011, 2016


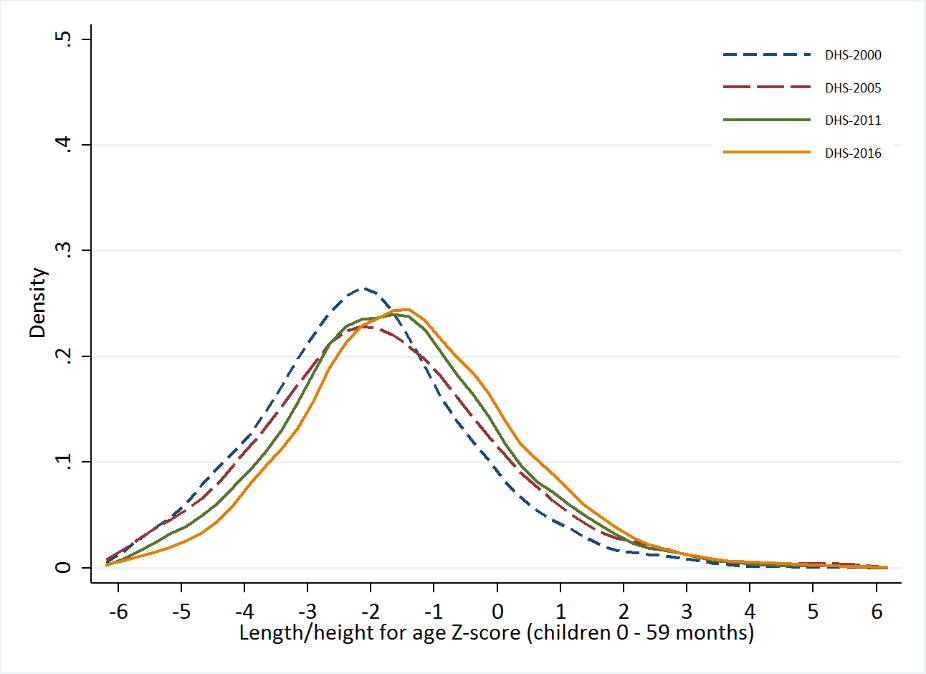


Source: Tasic et al, 2020 (Ethiopia stunting case study)

# Supplementary Appendix 9: Difference-in-difference analysis margins plot (Senegal Example)

## **Supplementary Appendix Figure 5:** Multivariable difference-in-difference results of maternal education predicting child HAZ in Senegal, 1992-2017


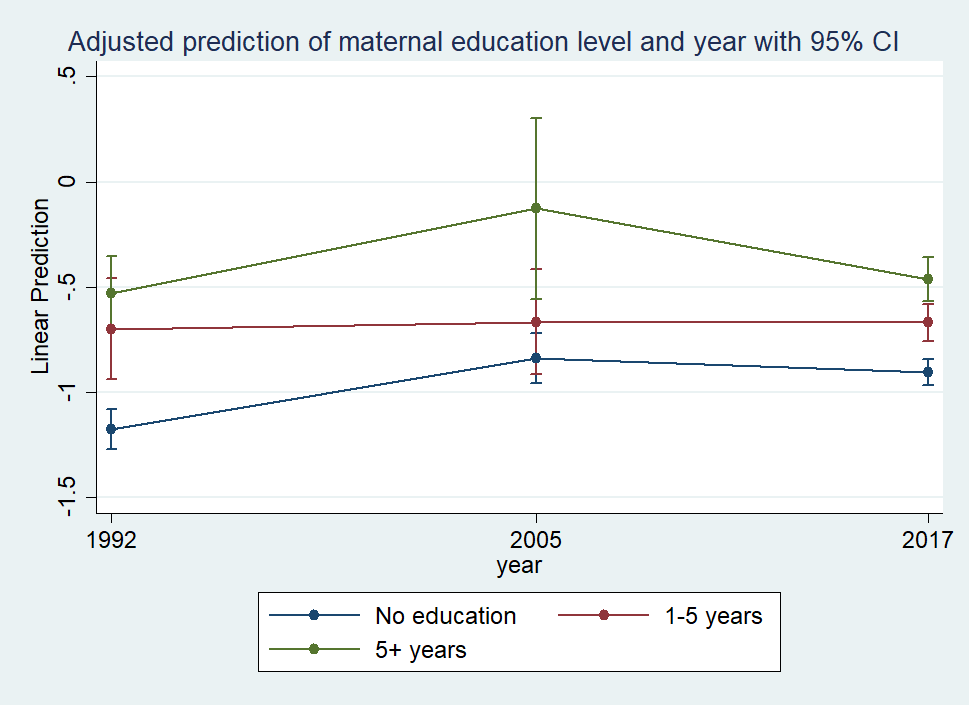


Source: Brar et al, 2020 (Senegal stunting case study)

# Supplementary Appendix 10: Qualitative Data Collection Tools (Ethiopia Example)

**In-depth Interview Guide for National Experts**

*Thank you for participating in this interview. The aim of this project is* to *conduct an in-depth assessment of the determinants, specifically contextual, underlying and immediate factors and relevant nutrition-specific and nutrition-sensitive policies and programs that may have driven stunting reduction in Ethiopia from 1990 to 2018. This interview aims to explore your perspective on Ethiopia’s nutrition evolution and is focused on stunting.*

Name: _________________________________________________________________________

Occupation: ___________________________________________________________________

Organization: _________________________________________________________________

Date of Interview: ____________________________________________________________

**Questions:**

*I would like to ask you a few questions about your background and work experience.*

1. What is your position and role?
2. Can you describe your experience in nutrition-specific and/or sensitive-programs?

**Overall trends in malnutrition & stunting**

You can introduce the discussion by explaining: *“I’d like to learn more about the status of stunting in Ethiopia.”*

1. How would you describe the trends in stunting in Ethiopia over the last 20 years?
2. *Probes: Do you think stunting has improved or worsened over the last twenty years, and why? Are these trends consistent nationally or are there any specific variations? Please identify regions with greatest/least improvements in stunting, and provide a reason.*

**Contextual/Basic Factors**

1. What sociopolitical factors do you think have influenced stunting?

- *Probes: Conflict, democratization, governance, decentralization, urbanization, factors relating to donors, etc.)*

1. What socioeconomic factors do you think have influenced stunting?

- *Probes: Poverty, wealth, labour migration/remittances, overall education, education of girls/women*

**Underlying Factors**

1. Can you describe changes in the health system over the last 20 years?

- *Probes: How has the accessibility or quality of health services changed? Availability of health providers? Health services provided? Financing/universal health coverage?*

1. How do you think these have influenced nutrition and stunting of children?
2. Can you describe the influence of environmental factors on stunting over the last two decades?

- *Probes: Environmental shocks, events such as drought/flood, climate, seasonality, etc.*

1. How do you think changes in agriculture have influenced children’s health and nutrition?

- *Probes: Types of crops produced, agricultural transition, employment, etc.*

1. What changes have you observed related to water, sanitation and hygiene? What do you think has contributed to these changes?

- *Probes: How has the accessibility to safe drinking water changed? Availability of toilets and sanitation facilities? Hygiene? Household crowding?*

**Immediate Factors**

1. How has dietary intake for mothers and children changed over the last 20 years?

- *Probes: Increased diversity? Micronutrient supplementation? Breastfeeding? Complementary feeding?*

1. How have other factors, such as fertility rates or spacing between pregnancies, changed over time? How do you think this has influenced chronic malnutrition?

**Policies & Programs**

*Provide the participant with the list of policies/programs. Give the respondent time to read the list. This list is meant to be a support for respondents. Individuals are not asked to speak about each policy/program. The first few questions are still broad/general to elicit perspectives on what the expert thinks are the key 3-5 policies/programs. If needed, the respondent can use the list to remind them of the policies/programs implemented.*

1. What nutrition-specific policies or programs do you feel have influenced stunting that have been implemented over the last 20 years?

- *Probe: Why do you think these policies/programs were important?*

1. What nutrition-sensitive policies or programs do you feel have influenced child malnutrition/stunting implemented over the last 20 years?

- *Probe: Why do you think these policies/programs were important?*
- *Probse: Education, WASH, RMNCH, poverty reduction (e.g., cash transfers),*

1. What are some of the contributions of these policies/programs/interventions to the reduction of chronic child undernutrition over the last 20 years?

- *Probe: What specific indicators/actions were taken as a result?*
- *Probe: Did any of these programs lead to the initiation/development of other efforts?*

*Note to Interviewer: For the main 3-5 policies/programs identified by the participant that have had an impact on stunting. Please say:*

*We have discussed several different policies and programs. It would be great to learn more about each of these policy efforts*

*For ____________________ (insert name of policy/program):*

1. Can you describe the policy/program?
2. What factors led to the introduction of this policy/program? Who initiated the policy change?
3. What were the main achievements of this policy/program? Were any objectives not achieved?
4. What was the coverage of the intervention (national, subnational, pilot, etc.)?
5. What were some of the facilitators to its implementation?
6. What were some of the barriers to its implementation?
7. Can you rank this policy/program/intervention on a scale from 1 (less important) to 10 (most important) to stunting reduction?
8. Based on the long list of policies/programs provided, can you think of any policies/programs/efforts that have not contributed to stunting reduction?

- *Probes: Why do you think this initiative wasn’t successful? (e.g., no impact on stunting reduction, scope, budget, poor implementation etc.), timeframe, contents, initiator/stakeholder?*

1. Are any key nutrition-specific or –sensitive policies or programs relevant to nutrition missing from this list?
2. Are there any other individuals (e.g., officials/practitioners/experts) knowledgeable about the stunting situation and stunting-reduction policies that you think we should speak to?

**In-Depth Interview Guide for Regional Key Informants**

*Thank you for participating in this interview. The aim of this project is* to *conduct an in-depth assessment of the determinants, specifically contextual, underlying and immediate factors and relevant nutrition-specific and nutrition-sensitive policies and programs that may have driven stunting reduction in Ethiopia from 2000 to 2018. This interview aims to explore your perspective on Ethiopia’s nutrition evolution and is focused on stunting.*

Types of local key informants that may be interviewed include:

1. Rural health post employees (e.g., physicians, nurses, midwives, etc.);
2. Community health workers;
3. Employees of kindergartens/schools;
4. Members of local women's councils;
5. Members of village health committees or local health structures;
6. Employees of any medical institutions working with children; and
7. Community leaders (traditional and religious leaders).

Name________________________________________________________________________

Village/woreda_____________________________________________________________

Occupation__________________________________________________________________

Organization________________________________________________________________

Age__________________________________________________ Gender________________

Contact information________________________________________________________

Date of interview___________________________________________________________

**Questions**

*I would like to ask you a few questions about your background and work experience.*

1. What is your current position and role?
2. Can you describe your experience in nutrition-specific and sensitive-programs?

**Overall trends in children nutrition**

You can introduce the discussion by explaining: *“I’d like to learn more about the status of chronic child undernutrition in your community.”*

1. How would you describe the trends in chronic undernutrition in this community over the last 20 years?
   1. *Probe: Have there been improvements or have things worsened?*

**Contextual factors**

1. What social or political factors do you think have influenced child malnutrition in this community?
   1. *Probes: Conflict, democratization, governance, decentralization, urbanization, factors relating to donors, etc.*
2. What socioeconomic factors do you think have influenced child malnutrition in this community?
   - *Probes: Poverty, wealth, labour migration/remittances, overall education, education of girls/women*

**Nutrition-specific & -sensitive policies and programs**

1. Over the past 15-20 years, what policies/programs do you think have improved children’s nutrition in the village/community? Who were they implemented by? Can you describe any results/changes achieved by these initiatives? What are the main challenges for implementing nutrition programs in this area (if they were implemented)?

**Underlying Factors**

1. How has accessibility and availability of health services changed over time in your village/region? What has caused these changes?
   1. *Probes: Number of health facilities, distance to health facilities, number of health workers (paid or volunteer), etc.*
2. What cultural or local factors do you feel have influenced knowledge about nutrition? Has this changed over the last 15-20 years?
3. How has the availability of food changed over time in your village/region? What has caused these changes?
   1. *Probes: Environmental changes, climate change, financial crisis, agricultural transition, etc.*
4. How has access to safe drinking water, toilet facilities or hygiene changed over time in this village/region? Why do you think these changes have occurred?
   1. *Probes: Where do you get your drinking water from? How do you treat the water?*
5. What are some of the main challenges for water, sanitation and hygiene in this community?

**Immediate Factors**

*Pregnancy, childbirth and breastfeeding*

You can introduce the discussion by explaining: *“I’d like to learn about nutrition related to pregnant women in this community.”*

1. What recommendations do you give to pregnant women regarding their nutrition to support the development of a fetus? Have these recommendations changed over time?
2. What recommendations do you give to women breastfeeding? Have these recommendations changed over time?
3. Do mothers often ask you about breastfeeding and general nutrition of children under-5 years? If not, where do they obtain most of their information on these issues?
4. What nutrition problems do women encounter during pregnancy and breastfeeding?
5. In your opinion, do mothers follow the recommendations of health providers regarding children’s health and nutrition?
6. How have other factors, such as fertility rates or spacing between pregnancies, changed over time? How do you think this has influenced chronic malnutrition?

*Nutrition of children under-5 years*

You can introduce the discussion by saying: *“Now I’d like to learn about the nutrition of children under-5 in this community.”*

1. How has dietary intake for mothers and children under-5 changed over the last 20 years?
   1. *Probes: Increased diversity? Micronutrient supplementation? Breastfeeding? Animal source protein?*
2. When do you advise the introduction of complementary food? What foods do you recommend?
3. What recommendations do you provide related to nutrition of children under-5 years?
4. What additional vitamins or supplements are provided to children in this community?
5. What do children under-5 years in your community typically eat?
   1. *Probe for childcare workers: What meals or food are provided to children in schools?*
6. How do you evaluate children’s daily diet? Do you think they get necessary vitamins for full growth (e.g., diverse diet, protein, fresh vegetables/fruits, etc.)? If not, why?
7. In your opinion, what are the main problems related to children’s nutrition and dietary intake?

**Focus Group Discussion Guide for Mothers in Communities**

**A. Focus Group Discussion Guide for Mothers in Communities (Children born 1987-1991)**

Region: ____________________________________________

Woreda: ____________________________________________

Date: ___________________________________________

Time of the beginning of the FGD: ______________________________

All of the participants in your focus group are mothers of children born between **1987–1991**. It is important to emphasize that this discussion is primarily related to their child born during this period. As these women might have other children, you should clarify to ensure they are speaking about their experiences related to their child born during the specific period of interest.

For this part, you will have list of mothers who are currently participating in the FGD. You can refer to the child born during this time period as the “index child”, while asking about nutrition, pregnancy and childbirth experiences.

**List names and information of participants**

| **No.** | **Name of a mother** | **Age/Age of Child** |
| --- | --- | --- |
| 1 |  |  |
| 2 |  |  |
| 3 |  |  |
| 4 |  |  |
| 5 |  |  |
| 6 |  |  |
| 7 |  |  |
| 8 |  |  |
| 9 |  |  |
| 10 |  |  |

Introduce this section by saying: “*Please remember that we are only talking about your experiences related to nutrition of your child born during 1987-1991”*

**General trends in children nutrition**

1. In your opinion, over the past 20 years, has children's nutrition in this village improved or worsened? What differences have you observed? Why do you think this has happened?

**Contextual factors**

1. What do you think are the reasons for the changes in children’s nutrition in your community? *Probe: Conflict (if applicable), reduction of poverty, improvements in quality of life, labour migration/remittances, changes in agriculture, urbanization, education (overall and among girls/women), women’s empowerment, etc.*

**Nutrition-specific and –sensitive policies & programs**

1. Over the last 15-20 years, what programs have been implemented to improve children’s nutrition in your village/community? Who were they implemented by? Can you describe results/changes achieved by these initiatives?

**Underlying Factors**

1. How has the availability of food changed over time in your village/region? What do you think has caused these changes
   - *Probe: Environmental changes, climate change, financial crisis, agricultural transition, etc.*
2. How has your ability to access care at health facilities changed over time in your community? What do you think has changed?
   - *Probes: Has the number of health workers (e.g., physicians, nurses, midwives, volunteers) improved over time? What about the types of services provided? Quality of services? Cost of services?*
3. Does your household have access to safe drinking water? If yes, when did you get it? Did you have access to clean drinking water in the 1990s?
   - *Probe: Where did you used to get your water for drinking?*
4. Does your household have access to toilet facilities? If yes, when did you get it? Did you have access to clean drinking water in the 1990s?
5. At what age did your children begin to wash their hands on their own?
6. What are some of the main challenges for water, sanitation and hygiene in this community?
7. What cultural or local factors do you feel have influenced local knowledge about nutrition? Has this changed over the last 15 years?

**Immediate factors**

This is a suggested opening statement that can be modified as needed: *Now, I would like to ask you about your pregnancy, childbirth and breastfeeding experiences with your child born during 1987-1991”.*  It is recommended to encourage mothers to respond freely. They can emphasize whatever they feel is important. If specific questions are already covered in the discussion, there is no need to bring them up. However, if they are not, then probe for details.

*Pregnancy, childbirth and breastfeeding*

1. During your pregnancy, how often did you visit the health worker and what advice did the health workers give about your diet?
2. Who helped you with the delivery of your child? *(Probe: health worker, nurse, family, friends, neighbors, traditional healers, etc.)?*
3. Remember the first days after giving birth. What recommendations for breastfeeding were given by health workers?
4. Did you immediately put your child to breastfeed? Why or why not?
5. Did you give any liquid or food to your child before first breastfeeding and why?
6. How was your child’s health during the first years of its life?
7. In the first year of your child's life, how often did you visit a health worker? What recommendations did you receive regarding the nutrition of the child?
8. Did you have any problems with breastfeeding, if so, what kind?
9. Was your child immunized?
10. How many months did you breastfed your children? Who advised you about the length of breastfeeding?

*Nutrition of children under-5 years*

1. When did you start giving complementary food? What did you give?
2. What foods did you feed your children? Were any foods forbidden to your child, and why? Did you prepare a separate meal?
3. How many times a day did you feed your child/children under-5 years?
4. Whose recommendations did you listen to regarding nutrition for your child?
5. Did you give vitamins or supplements in addition to meals?
6. Do you feel that the nutrition of your child was adequate and varied for growth?
7. Overall, in your community do you think that the nutrition of children has improved and become more diverse? Explain why or why not?
8. Did you ever go to a health facility for concerns such as nausea, diarrhea and other similar issues for you or your children? What diagnosis was made?

*This was the last question, if you have any thoughts, information, memories that you consider important, you can share!*

*Time of the end of FGI _________________________*

**B. Focus Group Discussion Guide for Mothers in Communities (Children born 1995-1999)**

Region: ____________________________________________

Woreda: ____________________________________________

Date: ___________________________________________

Time of the beginning of the FGD: ______________________________

All of the participants in your focus group are mothers of children born between **1995-1999**. It is important to emphasize that this discussion is primarily related to their child born during this period. As these women might have other children, you should clarify to ensure they are speaking about their experiences related to their child born during the specific period of interest.

For this part, you will have list of mothers who are currently participating in the FGD. You can refer to the child born during this time period as the “index child”, while asking about nutrition, pregnancy and childbirth experiences.

**List names and information of participants**

| **No.** | **Name of a mother** | **Age/Age of Child** |
| --- | --- | --- |
| 1 |  |  |
| 2 |  |  |
| 3 |  |  |
| 4 |  |  |
| 5 |  |  |
| 6 |  |  |
| 7 |  |  |
| 8 |  |  |
| 9 |  |  |
| 10 |  |  |

Introduce this section by saying: “*Please remember that we are only talking about your experiences related to nutrition of your child born during 1995-1999.”*

**General trends in children nutrition**

1. In your opinion, over the past 10-15 years, has children's nutrition in this village improved or worsened? What differences have you observed? Why do you think this has happened?

**Contextual factors**

1. What do you think are the reasons for the changes in children’s nutrition in your community? *Probe: Conflict (if applicable), reduction of poverty, improvements in quality of life, labour migration/remittances, changes in agriculture, urbanization, education (overall and among girls/women), women’s empowerment, etc.*

**Nutrition-specific and –sensitive policies & programs**

1. Over the last 10-15 years, what programs have been implemented to improve children’s nutrition in your village/community? Who were they implemented by? Can you describe results/changes achieved by these initiatives?

**Underlying Factors**

1. How has the availability of food changed over time in your village/region? What do you think has caused these changes
   - *Probe: Environmental changes, climate change, financial crisis, agricultural transition, etc.*
2. How has your ability to access care at health facilities changed over time in your community? What do you think has changed?
   - *Probes: Has the number of health workers (e.g., physicians, nurses, midwives, volunteers) improved over time? What about the types of services provided? Quality of services? Cost of services?*
3. Does your household have access to safe drinking water? If yes, when did you get it? Did you have access to clean drinking water in the late 1990s?
   - *Probe: Where did you used to get your water for drinking?*
4. Does your household have access to toilet facilities? If yes, when did you get it? Did you have access to clean drinking water in the late 1990s?
5. At what age did your children begin to wash their hands on their own?
6. What are some of the main challenges for water, sanitation and hygiene in this community?
7. What cultural or local factors do you feel have influenced local knowledge about nutrition? Has this changed over the last 15 years?

**Immediate factors**

This is a suggested opening statement that can be modified as needed: *Now, I would like to ask you about your pregnancy, childbirth and breastfeeding experiences with your child born during 1995 to 1999”.*  It is recommended to encourage mothers to respond freely. They can emphasize whatever they feel is important. If specific questions are already covered in the discussion, there is no need to bring them up. However, if they are not, then probe for details.

*Pregnancy, childbirth and breastfeeding*

1. During your pregnancy, how often did you visit the health worker and what advice did the health workers give about your diet?
2. Who helped you with the delivery of your child? *(Probe: Health worker, nurse, family, friends, neighbors, traditional healers, etc.)?*
3. Remember the first days after giving birth. What recommendations for breastfeeding were given by health workers?
4. Did you immediately put your child to breastfeed? Why or why not?
5. Did you give any liquid or food to your child before first breastfeeding and why?
6. How was your child’s health during the first years of its life?
7. In the first year of your child's life, how often did you visit a health worker? What recommendations did you receive regarding the nutrition of the child?
8. Did you have any problems with breastfeeding, if so, what kind?
9. Was your child immunized?
10. How many months did you breastfed your children? Who advised you about the length of breastfeeding?

*Nutrition of children under-5 years*

1. When did you start giving complementary food? What did you give?
2. What foods did you feed your children? Were any foods forbidden to your child, and why? Did you prepare a separate meal?
3. How many times a day did you feed your child/children under-5 years?
4. Whose recommendations did you listen to regarding nutrition for your child?
5. Did you give vitamins or supplements in addition to meals?
6. Do you feel that the nutrition of your child was adequate and varied for growth?
7. Overall, in your community do you think that the nutrition of children has improved and become more diverse? Explain why or why not?
8. Did you ever go to a health facility for concerns such as nausea, diarrhea and other similar issues for you or your children? What diagnosis was made?

*This was the last question, if you have any thoughts, information, memories that you consider important, you can share!*

*Time of the end of FGI _________________________*

**C. Focus Group Discussion Guide for Mothers in Communities (Children born 2011-2015)**

Region: ____________________________________________

Woreda: ____________________________________________

Date: ___________________________________________

Time of the beginning of the FGD: ______________________________

All of the participants in your focus group are mothers of children born between **2011-2015**. It is important to emphasize that this discussion is primarily related to their child born during this period. As these women might have other children, you should clarify to ensure they are speaking about their experiences related to their child born during the specific period of interest.

For this part, you will have list of mothers who are currently participating in the FGD. You can refer to the child born during this time period as the “index child”, while asking about nutrition, pregnancy and childbirth experiences.

**List names and information of participants**

| **No.** | **Name of a mother** | **Age/Age of Child** |
| --- | --- | --- |
| 1 |  |  |
| 2 |  |  |
| 3 |  |  |
| 4 |  |  |
| 5 |  |  |
| 6 |  |  |
| 7 |  |  |
| 8 |  |  |
| 9 |  |  |
| 10 |  |  |

Introduce this section by saying: “*Please remember that we are only talking about your experiences related to nutrition of your child born during 2011 to 2015”*

**General trends in children nutrition**

1. In your opinion, over the past 5 years, has children's nutrition in this village improved or worsened? What differences have you observed? Why do you think this has happened?

**Contextual factors**

1. What do you think are the reasons for the changes in children’s nutrition in your community? *Probe: Conflict (if applicable), reduction of poverty, improvements in quality of life, labour migration/remittances, changes in agriculture, urbanization, education (overall and among girls/women), women’s empowerment, etc.*

**Nutrition-specific and –sensitive policies & programs**

1. Over the last 5 years, what programs have been implemented to improve children’s nutrition in your village/community? Who were they implemented by? Can you describe results/changes achieved by these initiatives?

**Underlying Factors**

1. How has the availability of food changed over time in your village/region? What do you think has caused these changes
   - *Probe: Environmental changes, climate change, financial crisis, agricultural transition, etc.*
2. How has your ability to access care at health facilities changed over time in your community? What do you think has changed?
   - *Probes: Has the number of health workers (e.g., physicians, nurses, midwives, volunteers) improved over time? What about the types of services provided? Quality of services? Cost of services?*
3. Does your household have access to safe drinking water? If yes, when did you get it?
   - *Probe: Where did you used to get your water for drinking?*
4. Does your household have access to toilet facilities? If yes, when did you get it?
5. At what age did your children begin to wash their hands on their own?
6. What are some of the main challenges for water, sanitation and hygiene in this community?
7. What cultural or local factors do you feel have influenced local knowledge about nutrition? Has this changed over the last 15 years?

**Immediate factors**

This is a suggested opening statement that can be modified as needed: *Now, I would like to ask you about your pregnancy, childbirth and breastfeeding experiences with your child born during 2011 to 2015”.*  It is recommended to encourage mothers to respond freely. They can emphasize whatever they feel is important. If specific questions are already covered in the discussion, there is no need to bring them up again. However, if they are not, then please probe for details.

*Pregnancy, childbirth and breastfeeding*

1. During your pregnancy, how often did you visit the health worker and what advice did the health workers give about your diet?
2. Who helped you with the delivery of your child? *(Probe: Health worker, nurse, family, friends, neighbors, traditional healers, etc.)?*
3. Remember the first days after giving birth. What recommendations for breastfeeding were given by health workers?
4. Did you immediately put your child to breastfeed? Why or why not?
5. Did you give any liquid or food to your child before first breastfeeding and why?
6. How was your child’s health during the first years of its life?
7. In the first year of your child's life, how often did you visit a health worker? What recommendations did you receive regarding the nutrition of the child?
8. Did you have any problems with breastfeeding, if so, what kind?
9. Was your child immunized?
10. How many months did you breastfed your children? Who advised you about the length of breastfeeding?

*Nutrition of children under-5 years*

1. When did you start giving complementary food? What did you give?
2. What foods did you feed your children? Were any foods forbidden to your child, and why? Did you prepare a separate meal?
3. How many times a day did you feed your child/children under-5 years?
4. Whose recommendations did you listen to regarding nutrition for your child?
5. Did you give vitamins or supplements in addition to meals?
6. Do you feel that the nutrition of your child was adequate and varied for growth?
7. Overall, in your community do you think that the nutrition of children has improved and become more diverse? Explain why or why not?
8. Did you ever go to a health facility for concerns such as nausea, diarrhea and other similar issues for you or your children? What diagnosis was made?

*This was the last question, if you have any thoughts, information, memories that you consider important, you can share!*

*Time of the end of FGI _________________________*
